# Using Smartphone-Based Psychoeducation to Reduce Postnatal Depression Among First-Time Mothers: Randomized Controlled Trial

**DOI:** 10.2196/12794

**Published:** 2019-05-14

**Authors:** Ko Ling Chan, Wing Cheong Leung, Agnes Tiwari, Ka Lun Or, Patrick Ip

**Affiliations:** 1 Department of Applied Social Sciences The Hong Kong Polytechnic University Hong Kong China (Hong Kong); 2 Department of Obstetrics & Gynaecology Kwong Wah Hospital Hong Kong China (Hong Kong); 3 School of Nursing Hong Kong Sanatorium & Hospital Limited Hong Kong China (Hong Kong); 4 Department of Industrial and Manufacturing Systems Engineering The University of Hong Kong Hong Kong China (Hong Kong); 5 Department of Paediatrics and Adolescent Medicine The University of Hong Kong Hong Kong China (Hong Kong)

**Keywords:** smartphone technology, postnatal depression, psychoeducation, randomized controlled trial

## Abstract

**Background:**

Smartphone-based psychoeducation interventions may be a low-cost, user-friendly alternative to resource-consuming, face-to-face antenatal classes to educate expectant mothers.

**Objective:**

This study aimed to empirically examine whether such an intervention would lead to reduced postnatal depression, anxiety, or stress and result in a better health-related quality of life.

**Methods:**

A single-blind randomized controlled trial was conducted in Hong Kong. All first-time expectant mothers with less than 24 weeks of gestation remaining and attending the antenatal clinic at a public hospital were included. Participants were assigned to the intervention group or the control group by drawing lots. The lots, presented in sealed opaque envelopes, were randomly designated as “intervention” or “control” by stratified randomization. The intervention, a psychoeducational mobile app, was provided in addition to the treatment as usual (TAU) services from the hospital. Follow up with participants took place at 4 weeks postpartum. The primary outcome was the difference in the levels of antenatal and postnatal depression, assessed by the Edinburgh Postnatal Depression Scale (EPDS). The intention-to-treat approach was employed in the analyses.

**Results:**

The final sample was 660 expectant mothers (n_intervention_=330 and n_control_=330). The mean difference in EPDS scores between the two groups was −0.65 (95% CI −1.29 to 0.00; *P*=.049) after adjusting for confounding factors. Associations were found between participation in the intervention and reduced depression, and attendance in TAU classes and increased stress levels.

**Conclusions:**

The smartphone-based intervention plus TAU services was effective in reducing postnatal depression at 4 weeks postpartum compared with a control condition of TAU only, making this a cost-effective alternative to TAU education for expectant mothers. Limitations of the study included the short postpartum period after which the follow-up assessment was conducted and the inclusion of first-time mothers rather than all mothers.

**Trial Registration:**

HKU Clinical Trials Registry HKUCTR-2024; http://www.hkuctr.com/Study/Show/ 34f62a2f6d594273a290491827206384

## Introduction

### Background

The potential positive effects of engaging parents in parental and infant health have increased research interest globally. Antenatal education classes, which provide an opportunity to teach expectant mothers about known complications and the support needed during pregnancy and childbirth, are effective for preparing expectant couples for parenthood. Research has revealed that antenatal classes are useful for reducing postnatal distress among first-time mothers (eg, at 6 weeks postpartum in a study by Matthey et al [1]), reducing the risks of postnatal depression (PND) [2], increasing mothers’ awareness of pain relief techniques [3], and reducing parental stress and anxiety symptoms [4]. Among these negative health consequences, PND affects approximately 13% of the population as found in a meta-analysis by O'hara and Swain [5]. Review studies have observed that an antenatal intervention can be effective for preventing PND and, in turn, prevent future negative consequences for the mother, child, and family [6].

Despite the well-documented effects of such traditional antenatal interventions, the amount of face-to-face support available for expectant parents is often limited, owing to a lack of resources, social support, and personnel [7]. To face the increasing burdens of the health care system in the past decade, advanced computer-based technologies have been considered as effective means of providing support to health care consumers, given their popularity and easy accessibility [8]. More specifically, smartphone technology has been increasingly demonstrated as a useful tool to disseminate health information, by providing psychoeducation and/or timely interactions via short message service text messaging or two-way communication between patients and health professionals [9]. There has been evidence showing that therapist audio recordings and remote communication between patients and service providers offered in mobile apps are flexible, portable, and relatively low cost, making them an ideal choice for patients who are restricted by time and location constraints and cannot partake in traditionally used services [10]. Recent empirical research has also shown its effectiveness in psychoeducation, skills training, and two-way communication with health professionals via smartphone apps to promote well-being among mental health patients [1], and meta-analytic studies have also demonstrated that smartphone-based interventions may reduce depression and anxiety [11,12].

### Objectives of the Study

Due to the previous successful application of smartphone technology in health care, the aim of the present study was to test the effectiveness of a smartphone-based intervention to educate expectant parents during pregnancy. A randomized controlled trial (RCT) was conducted to determine if a smartphone-based intervention can be a low-cost alternative to traditional face-to-face antenatal classes for educating expectant parents and promoting health outcomes. Compared with treatment as usual (TAU) where approximately 50% of the expectant parents could be enrolled in a nurse-led antenatal class while the remainder would receive information booklets in print form, a smartphone-based intervention can deliver all essential materials that would be taught in class. Materials comprised small video clips and short passages for parents to read or watch at their convenience. Parents could also interact with health professionals using the specifically designed platform whenever they had an inquiry. The intervention was expected to provide parenthood education without the time or space constraints. With better preparation, expectant parents were hypothesized to show lower levels of PND as well as postnatal anxiety and stress postintervention versus those who received only TAU or standard antenatal services in hospitals. In addition, this study also explored the overall effects of this smartphone-based intervention versus traditional TAU classes on promoting postnatal health and reducing maternal PND, anxiety, and stress.

## Methods

### Study Design

A single-blind RCT was conducted to compare an intervention group, where participants used a smartphone-based app for antenatal education, and a control TAU group that received antenatal services from Kwong Wah Hospital (KWH), a major public hospital in Hong Kong, during 2017 and 2018. This study obtained ethical approval from the Institutional Review Board of the Hospital Authority (Kowloon West Cluster) Research Ethics Committee and was registered under the Clinical Trials Registry of Clinical Trials Centre of the University of Hong Kong. No important changes to methods and outcomes were made after the commencement of the study.

### Participants

First-time pregnant women with less than 24 weeks of gestation remaining and who attended the antenatal clinic at KWH were recruited to participate in the study. To ensure the representativeness of the sample, no limit was set on the ethnicity or nationality of the participants. Eligible participants were first-time expectant mothers receiving regular antenatal care services at KWH, were able to read and understand Chinese or English, and were willing to consent to the terms of the study. The nursing staff at KWH provided assistance in the identification of potential participants during the recruitment procedure. Expectant mothers were excluded if they were unable to give informed written consent or communicate with the interviewers. The participation of their partners was encouraged but was not a requirement for participation.

### Randomization and Masking

Participants were assigned to the intervention or control group using lots in a ratio of 1:1. The lots, presented in sealed opaque envelopes, were randomly designated as “intervention” or “control” by stratified randomization, with random numbers generated from statistical software. Each participant received a sealed envelope with information indicating the group they were assigned to. The recruitment and randomization procedures were conducted by different groups of researchers, and the outcome assessors were blinded to the allocation in each group.

### Intervention and Control

The intervention involved the provision of access to a smartphone app, the iParent app (hereinafter referred to as the app), to the selected expectant mothers (n=330) between their first visit to the antenatal clinic and childbirth in addition to the TAU services they received at KWH. All materials presented in the app were equivalent to those offered in the face-to-face nurse-led antenatal TAU classes at KWH. For example, there were articles about nutrition, infant caring, and vaccine injections for infants, as well as videos demonstrating what expectant mothers might face when delivering their baby. The major difference was the mode of information delivery: information was delivered off-site, organized by topic to increase usability, and open to access at any time and anywhere for assigned users. Furthermore, a platform within the app allowed users to ask questions related to pregnancy, childbirth, and infant health and care. All questions were answered by obstetricians via private, direct messages within the app and then shared in the Frequently Asked Questions module of the app, if permitted by the user and after personal identifiable information was removed. This function dealt only with regular and nonemergent consultations.

The app was designed by the authors and was tested and updated through various trials. For the intervention group, they were given a specific login name and password (which participants were encouraged to change when using the app for the first time) for registration and were permitted free access to the app. Use parameters such as the frequencies of logins and the time spent using the app were recorded. Prompts would be made via emails if the participants in the intervention group did not log in to the app during the study period.

The control group (n=330) received standard services provided by KWH only. They could enroll in the 4-session, nurse-led antenatal classes. The control group participants were not allowed to access the app.

### Procedure

At their first visit to the antenatal clinic at KWH, eligible participants were informed about the study details and participant rights in a private room in the hospital. In particular, they were ensured that refusal to participate would not affect any service they received at KWH. Eligible expectant mothers who agreed to participate provided written consent and completed the baseline T1 survey that assessed their antenatal depression, anxiety and stress levels, health-related quality of life (QoL), and demographic characteristics. Participants also indicated their preferred methods of contact at later stages of the study.

Around 4 weeks after childbirth, participants were contacted for the follow-up T2 survey. The follow-up T2 survey included scales assessing their PND, anxiety and stress levels, health-related QoL, and usage of the app and antenatal classes provided by the hospital.

Although there was no potential risk associated with the intervention, medical and health professionals of the antenatal clinic at KWH were available for assistance during the RCT.

### Outcomes

The primary outcome was the difference between the level of depression before and after the RCT (ie, depression during pregnancy and PND) among the expectant mothers. The validated Chinese version of the 10-item Edinburgh Postnatal Depression Scale (EPDS) was used to detect depressive symptoms [13]. Total scores ranged from 0 to 30, with a higher score indicating more severe depression levels. The Cronbach alphas of the Chinese EPDS in this study were .85 at baseline survey and .84 at follow up, indicating a good reliability.

Secondary outcomes included differences in anxiety levels, stress levels, and health-related QoL before and after the RCT. Anxiety and stress levels were assessed with the Anxiety and Stress subscales of the Depression Anxiety Stress Scale (DASS) [14]. Each subscale has 7 items. Total scores ranged from 0 to 21, with higher scores indicating higher levels of anxiety or stress. The Cronbach alphas of the Anxiety subscale were .78 and .74 at baseline and follow up, respectively, whereas those of the Stress subscale were .85 and .83 at the 2 time points, respectively. Health-related QoL was measured by the 12-item Short Form Health Survey (SF-12) [15]. Scores on the SF-12 were computed as 2 separate composite scores: one for Physical Component Summary (PCS) and the other for Mental Component Summary (MCS). Both PCS and MCS ranged from 0 to 100, with higher scores indicating better health-related QoL. The reliability of SF-12 was good in this study, with Cronbach alphas of .87 and .85 at baseline and follow up, respectively.

We also recorded selected demographic characteristics and antenatal service utilization among the participants. These demographic characteristics included the following: age, education level, employment status, marital status, household income, and presence of diagnosed chronic health conditions or mental illness. The use of the app and other relevant services (eg, antenatal classes and other resources about pregnancy such as books and websites) was documented by self-reported responses from study participants (ie, through the use of yes/no questions).

### Statistical Analysis

All statistical analyses were conducted and underscored by the intention-to-treat principle. All missing data were treated by the last observation carried forward imputation method. All statistical analyses were conducted with IBM SPSS 24.0 using a significance level of .05.

With reference to a previous psychological intervention for 6 weeks PND [16], a sample size of at least 276 participants was required for both the intervention and control groups in this study. The final sample in this study was 330 participants in each group.

The analysis of covariance (ANCOVA) was used to test the hypotheses of this study. In particular, the effectiveness of the intervention was first examined by comparing the primary outcomes between groups, adjusted for demographic characteristics and the use of the app and relevant antenatal services. Similar analyses were also conducted on the secondary outcomes.

The effects of the intervention and the attendance of antenatal classes were then tested using the ANCOVA, which compared primary and secondary outcomes, adjusted for demographics, service usage, and baseline T1 scores.

This RCT was registered with the HKU Clinical Trials Registry (HKUCTR-2024).

### Role of the Funding Source

The funder of this study had no role in the study design, data collection, data analysis, data interpretation, or writing of the report. The corresponding author had full access to all the data in this study and had final responsibility for the decision to submit for publication.

## Results

**Figure 1:**
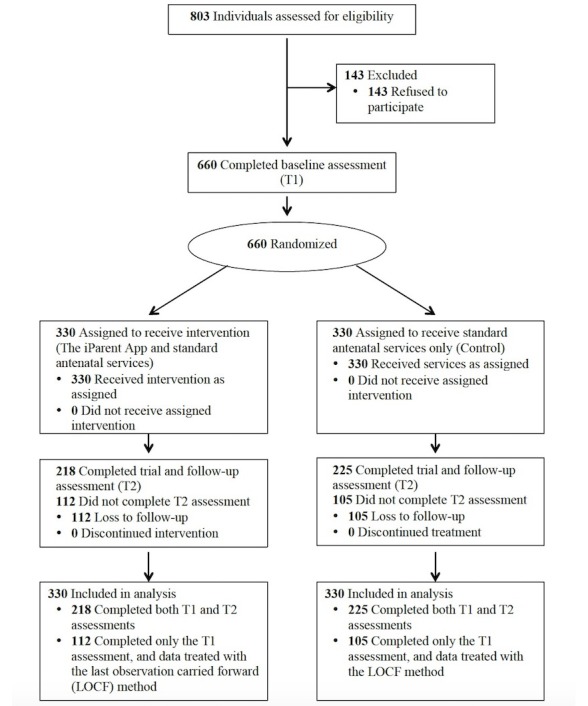
Consolidated Standards of Reporting Trials flow diagram of the randomized controlled trial.

In the recruitment stage, 803 first-time expectant mothers were assessed for eligibility and 660 were included for the baseline T1 survey and randomization (response rate=82.2%). Of the 660 participants, 330 were randomly assigned to the intervention group and 330 were allocated to the control group. At the follow-up T2 survey after the intervention, the retention rates were 66.1% for the intervention group and 68.2% for the control group. More details on the sampling procedures are presented in the Consolidated Standards of Reporting Trials flow diagram (see Figure 1). No visible harm or unintended effect was noted in the groups. There was no statistically significant difference in the demographic characteristics between the participants who completed the study and those who had withdrawn (all *P*>.05).

Demographic characteristics of the participants at baseline are shown in Table 1. All participants were Chinese. The mean ages of the intervention group and control group were 31.3 years (SD 4.6) and 31.2 years (SD 4.5), respectively. There was no significant between-group difference in any demographic characteristic measured in this study (all *P*>.05).

Table 2 presents the mean scores, standard deviation (SD), internal reliabilities, and results of the between-group comparisons of the primary and secondary outcomes (N=660). The mean EPDS score of the intervention group dropped from 7.3 (SD 4.6) to 5.3 (SD 4.4) after the RCT and that of the control group dropped from 7.2 (SD 4.6) to 5.9 (SD 4.7) after receiving TAU care. The mean difference between groups was −0.65 (95% CI −1.29 to 0.00; *P*=.049), which was a significant difference after adjusting for the baseline T1 mean scores and demographic characteristics.

The results in this study did not show significant between-group differences in the secondary outcomes tested (ie, the mean scores of anxiety, stress, and health-related QoL before and after the RCT). Both of the mean scores on the Anxiety subscale and Stress subscale of the DASS dropped in both groups from baseline to follow up, although the differences between groups were not significant after the adjustment of the baseline scores and demographic factors (*P*=.94 and *P*=.74, respectively). Nonsignificant mean differences between groups were also found in the SF-12 scores, although the 2 composite scores increased from baseline to post-RCT follow up in both groups (*P*=.54 and *P*=.81, respectively).

**Table 1:** Selected demographic characteristics of the mothers.

Characteristics		Intervention (n=330)	Control (n=330)	*P* value
Age at baseline (years), mean (SD)		31.3 (4.6)	31.2 (4.5)	.84
**Educational attainment, n (%)**				.20
	Junior high school or below	27 (8.2)	41 (12.4)	.20
	High school	110 (33.3)	109 (33.0)	.20
	University, higher institution, or above	191 (57.9)	179 (54.2)	.20
	Missing	2 (0.6)	1 (0.3)	.20
Employed, n (%)		268 (81.2)	256 (77.6)	.28
**Marital status, n (%)**				.21
	Married	280 (84.8)	282 (85.5)	.21
	Living or cohabiting as couple	12 (3.6)	20 (6.1)	.21
	Single and never married	28 (8.5)	23 (7.0)	.21
	Separated, divorced, or widowed	1 (0.3)	2 (0.6)	.21
	Missing	9 (2.7)	3 (0.9)	.21
Diagnosed with chronic health conditions^a^, n (%)		17 (5.2)	24 (7.3)	.40
Diagnosed with mental illness, n (%)		2 (0.6)	0 (0)	.24
**Household income (monthly), n (%)**				.98
	HKD^b^ 15,000 (approximately US $1911) or below	46 (13.9)	48 (14.5)	.98
	HKD 15,001 to HKD 39,999 (approximately US $1911-$5097)	123 (37.3)	121 (36.7)	.98
	HKD 40,000 (approximately US $5097) or above	148 (44.8)	146 (44.2)	.98
	Missing	13 (3.9)	15 (4.5)	.98
	Receiving social security assistance	16 (4.8)	11 (3.3)	.22

^a^A condition that is long-lasting or recurrent, for example, diabetes mellitus, chronic obstructive pulmonary disease, asthma, heart failure, cancer, arthritis, and chronic renal failure.

^b^HKD: Hong Kong Dollar.

**Table 2:** Mean (SD) values of the outcome measures pre- (baseline, T1) and postintervention (follow up, T2) and the between-group mean differences.

Outcome	Cronbach alpha	Intervention (n=330), mean (SE)		Control (n=330), mean (SE)		Adjusted mean difference between groups^a^ (95% CI)	*P* value
		Baseline (T1)	Follow up (T2)	Baseline (T1)	Follow up (T2)		
Edinburgh Postnatal Depression Scale^a,b^	.84-.85	7.3 (4.6)	5.3 (4.4)	7.2 (4.6)	5.9 (4.7)	−0.65 (−1.29 to 0.00)	.049
DASS^c^ (Anxiety)^d^	.74-.78	3.1 (2.7)	1.9 (2.1)	2.8 (2.6)	1.8 (2.3)	0.01 (−0.30 to 0.32)	.94
DASS (Stress)^e^	.83-.85	3.9 (3.5)	3.0 (3.1)	3.8 (3.4)	2.9 (3.1)	0.07 (−0.35 to 0.50)	.74
SF-12^f,g^ (Physical Component Summary)	.85-.87	45.5 (7.0)	48.8 (6.9)	46.4 (7.0)	48.8 (7.2)	0.32 (−0.71 to 1.34)	.54
SF-12 (Mental Component Summary)	.85-.87	48.6 (8.9)	51.2 (8.4)	48.2 (8.9)	51.2 (9.0)	−0.15 (−1.39 to 1.09)	.81

^a^Estimated between-group difference (intervention – control) after giving birth (follow up, T2), adjusted for baseline (T1) values.

^b^Possible ranges from 0 to 30, with higher scores indicating more severe depressive symptoms.

^c^DASS: Depression Anxiety Stress Scale.

^d^Range: 0 to 21 (with higher scores indicating more severe anxiety symptoms).

^e^Range: 0 to 21 (with higher scores indicating more severe stress symptoms).

^f^SF-12: 12-Item Short Form Health Survey.

^g^Range: 0 to 100 (with higher scores indicating better health-related quality of life).

**Figure 2:**
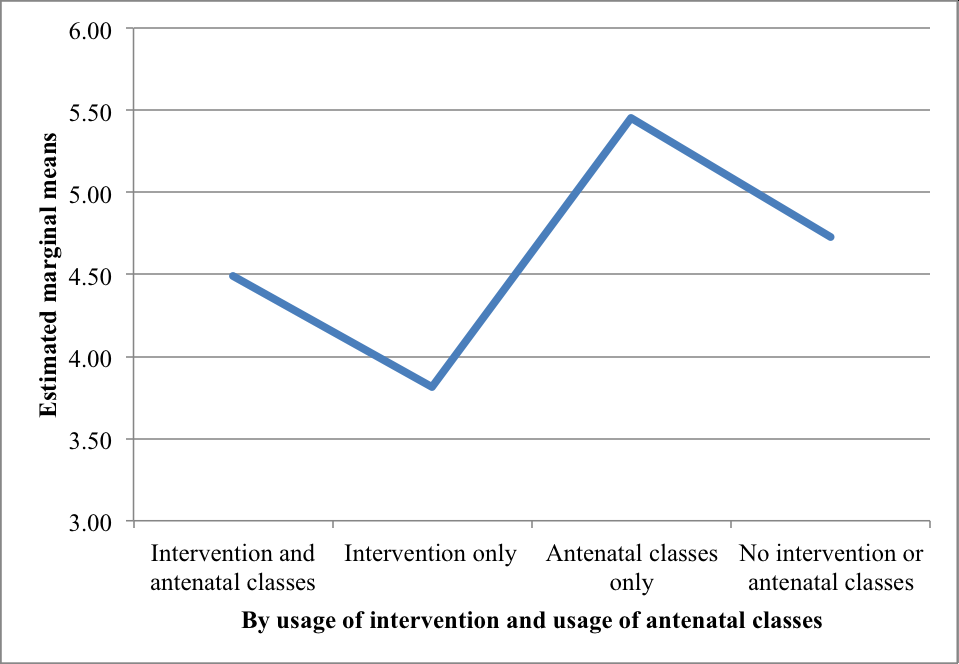
Estimated marginal means of the Edinburgh Postnatal Depression Scale scores postintervention (follow up, T2), adjusted for baseline (T1) scores by usage of intervention and antenatal classes.

Table 3 summarizes the findings on the effects of participation in the intervention and attendance in the nurse-led antenatal classes on the primary and secondary outcomes (N=443). Among these 443 participants who completed the whole study, 218 were in the intervention group and 225 were in the control group. Concerning the attendance in the antenatal classes, 272 of the 443 participants had enrolled in the classes whereas the remaining 171 had not. The mean scores of the EPDS were significantly different between the intervention group and control group (estimated marginal means for the intervention group was 4.2 [SE 0.3]; estimated marginal means for the control group was 5.1 [SE 0.3]; *P*=.01), with the follow-up scores of EPDS lower in the intervention group. Relevant results are noted in Figure 2. On the contrary, those of the Stress subscale of the DASS were significantly different between the participants who attended the nurse-led antenatal classes versus those who did not (estimated marginal means was 2.8 [SE 0.2] for those who attended and 1.9 [SE 0.2] for those who did not; *P*=.04). These findings demonstrated that participants who attended TAU classes reported higher levels of stress than those who did not attend the classes at follow up.

These findings indicated that participation in the intervention was significantly associated with decreased depression levels, whereas attending TAU nurse-led antenatal classes was significantly associated with increased stress levels after the study period.

**Table 3:** Mean (SD) values of the outcome measures pre- (baseline, T1) and postintervention (follow up, T2), and between-group differences between intervention and antenatal class participation.

Outcome and time of assessment		Usage of intervention, mean (SE)		Usage of antenatal classes, mean (SE)		*P* value^a^ (intervention)	*P* value^a^ (antenatal classes)
		Intervention (n=218)	Control (n=225)	Attended (n=272)	Not attended (n=171)		
**Edinburgh Postnatal Depression Scale^b^**							
	Baseline (T1)	7.3 (4.7)	7.0 (4.4)	7.2 (4.6)	7.1 (4.4)	.01	.47
	Follow up (T2)	4.3 (3.9)	5.2 (4.3)	5.0 (4.2)	4.3 (4.0)	—^c^	—
	Estimated marginal means (SE)	4.2 (0.3)	5.1 (0.3)	5.0 (0.3)	4.3 (0.3)	—	—
**DASS^d^ (Anxiety)^e^**							
	Baseline (T1)	3.2 (2.9)	2.7 (2.5)	3.1 (2.8)	2.7 (2.6)	.30	.06
	Follow up (T2)	1.4 (1.7)	1.1 (1.6)	1.4 (1.8)	1.0 (1.3)	—	—
	Estimated marginal means (SE)	1.3 (0.1)	1.1 (0.1)	1.4 (0.1)	1.0 (0.1)	—	—
**DASS (Stress)^f^**							
	Baseline (T1)	3.9 (3.4)	3.7 (3.4)	3.9 (3.4)	3.8 (3.4)	.99	.04
	Follow up (T2)	2.5 (2.7)	2.4 (2.8)	2.8 (2.9)	1.9 (2.5)	—	—
	Estimated marginal means (SE)	2.4 (0.2)	2.4 (0.2)	2.8 (0.2)	1.9 (0.2)	—	—
**SF-12^g^ (Physical Component Summary)^h^**							
	Baseline (T1)	45.9 (6.9)	46.6 (7.2)	46.3 (7.2)	46.2 (6.9)	.18	.81
	Follow up (T2)	50.9 (5.9)	50.0 (7.1)	50.6 (6.5)	50.1 (6.6)	—	—
	Estimated marginal means (SE)	50.8 (0.5)	50.0 (0.4)	50.6 (0.4)	50.2 (0.5)	—	—
**SF-12 (Mental Component Summary)^h^**							
	Baseline (T1)	48.3 (9.0)	48.8 (8.5)	48.9 (9.0)	48.1 (8.3)	.25	.63
	Follow up (T2)	52.1 (8.2)	53.2 (8.0)	52.0 (8.1)	53.7 (7.9)	—	—
	Estimated marginal means (SE)	52.3 (0.6)	53.3 (0.5)	52.0 (0.5)	53.6 (0.6)	—	—

^a^*P* values obtained with analysis of covariance, with baseline (T1) scores being adjusted.

^b^Range: 0 to 30 (with higher scores indicating more severe depressive symptoms).

^c^Not applicable.

^d^DASS: Depression Anxiety Stress Scales.

^e^Range: 0 to 21 (with higher scores indicating more severe anxiety symptoms).

^f^Range: 0 to 21 (with higher scores indicating more severe stress symptoms).

^g^SF-12: 12-Item Short Form Health Survey.

^h^Range: 0 to 100 (with higher scores indicating better health-related quality of life).

## Discussion

Our findings provide empirical support for the relative effectiveness of a smartphone-based intervention in reducing PND among first-time mothers at 4 weeks postpartum. Compared with those who received only standard TAU services, mothers who had received the intervention and used the iParent app in addition to standard care reported significantly lower levels of PND when baseline depression and other confounding factors were adjusted for. This finding compares favorably with previous research showing that computer- or smartphone-based technologies are effective in promoting user health [9-12,16,17]. More specifically, this study sheds light on the potential generalizability of the relative effectiveness of these technologies in reducing depression among patients’ diagnoses of mental illness to other health care service users such as pregnant women. Such smartphone-based technologies do not only help manage and/or monitor depressive symptoms among diagnosed patients but might also be useful for preventing or reducing depression among the general population.

Our findings also show that the intervention, more so than the traditional face-to-face TAU classes, was significantly associated with reduced levels of PND when adjusted for baseline depression during pregnancy. Through the iParent platform, expectant parents had essential information on antenatal care, postnatal care, infant care, and child protection at their fingertips. The portability and mobility of the app and the popularity of its use may indeed facilitate the engagement of parents in parenthood preparation with less time and space constraints [18], which may in turn promote parental health and a successful pregnancy, since asking questions or discussions with health professionals about relevant issues can be done easily through the app [7]. Another possible reason for the effectiveness of the smartphone-based intervention may be its provision of unlimited access to essential information; compared with the traditional face-to-face TAU classes that are normally one-off events, the app allowed for continuous access to information. A third possible mechanism for the effectiveness of the intervention might be the increase of a sense of connection among the mothers when they were using the app to interact with the health professionals. Normally, new mothers, who need time for physical recovery, might feel isolated for the first few weeks after giving birth, but iParent could provide them an opportunity to stay connected with others. This sense of connection might increase the level of perceived social support from professionals and even from peers (ie, other mother users of the app who could ask questions and share experiences in the app), which might in turn reduce PND [19].

The encouraging evidence found herein supports the relative effectiveness of iParent and warrants the potential use of other advanced technologies as viable low-cost alternatives to face-to-face TAU services in educating and monitoring the nonemergent cases of health care service users to reduce the burden to the health care system and relevant professionals.

This study observed reductions in postnatal anxiety and stress levels after the intervention, although the differences were not statistically significant. This diverges from the results shown in a meta-analysis by Firth et al [12]. Our preliminary results indicated that the smartphone-based intervention might not be sufficiently effective to add extra value on top of the standard TAU services in reducing anxiety and stress among first-time mothers. However, further empirical evidence is needed before establishing a conclusion. Indeed, researchers have suggested that the design of the RCT itself might affect the results obtained. For example, effect sizes of the smartphone-based interventions are found to be greater when compared with waitlist controls than active controls [12], of which the latter condition was employed in this RCT.

A surprising finding in this study was the association noted between attendance of face-to-face antenatal classes and levels of postnatal stress. After adjustment for baseline stress, first-time mothers who had attended the classes reported higher levels of postnatal stress than did those who did not attend any. This discovery adds some evidence to the claim that the effects of TAU antenatal classes might not be universal across all populations. For example, a Spanish study found increased levels of stress during childbirth among immigrants but decreased levels among a local sample after attending antenatal classes [20]. It was suggested that the group receiving antenatal classes may have increased awareness of problems and difficulties that may arise during the postnatal period, making them more likely to show higher stress levels [21]. Other researchers have suggested that differences across individuals might be due to gender, age, education level, and the different expectations about participating in the classes [22]. Future studies should assess expectant parents’ expectations on the education they receive either in such classes or through the intervention as a covariate to further explore the underlying effect of the program on parental stress.

To our knowledge, this is the first RCT study of a smartphone-based psychoeducation intervention for first-time expectant mothers. However, the study has several limitations, and the present findings should not be generalized unequivocally. First, these outcomes were measured up to 4 weeks postpartum, and outcomes during childbirth were not assessed. It is possible that these effects might change across time, so future studies should extend the study period to include more precise longitudinal tracking of such outcomes. Second, this study only analyzed health outcomes among first-time mothers. Health outcomes of fathers and infants are also useful indicators of the effectiveness of these kinds of interventions for pregnancy and childbirth. Thus, future research should also include an assessment of paternal and child outcomes. Third, similar to studies involving self-reported data, our study is subjective to reporting bias and recall error. Future research could employ more and/or complementary objective assessments such as journals, secondary clinical data, and obstetricians’ reports. Finally, this study only employed first-time mothers residing in Hong Kong, and previous local research has suggested the possibility of ethnic differences in the effectiveness of antenatal education on health outcomes [20]. Future research may extend such RCT initiatives to other populations in the world to offset cultural bias.

Using an RCT, the conclusions of this study suggest that smartphone-based psychoeducation plus standard antenatal services at hospitals in Hong Kong can be effective in reducing PND at 4 weeks postpartum among first-time mothers when compared with a control group receiving standard services only.

## Abbreviations

ANCOVA: analysis of covariance
DASS: Depression Anxiety Stress Scale
EPDS: Edinburgh Postnatal Depression Scale
KWH: Kwong Wah Hospital
MCS: Mental Component Summary
PCS: Physical Component Summary
PND: postnatal depression
QoL: quality of life
RCT: randomized controlled trial
SF-12: 12-item Short Form Health Survey
TAU: treatment as usual
